# Radiology in the age of artificial intelligence: challenges and opportunities

**DOI:** 10.1590/0100-3984.2024.57.e1-en

**Published:** 2024-07-12

**Authors:** Tulio Augusto Alves Macedo, Manoel de Souza Rocha

**Affiliations:** 1 School of Medicine, Universidade Federal de Uberlândia (UFU), Uberlândia, MG, Brazil. Email: tuliomacedo@ufu.br; 2 Department of Radiology and Oncology, Faculdade de Medicina da Universidade de São Paulo (FMUSP), São Paulo, SP, Brazil

In an era marked by unprecedented technological advances, artificial intelligence (AI) is emerging as a transformative force in many areas, including the field of medicine.

Recently, **Radiologia Brasileira** featured an interesting article that put the capabilities of ChatGPT to the test in a specialist examination promoted by the Brazilian College of Radiology and Diagnostic Imaging^(1)^. ChatGPT is an advanced language model, capable of generating text and responses, interacting with humans in natural language.

Despite not having achieved an overall passing score on the radiology exam, ChatGPT produced a result that was surprisingly close to the necessary threshold. The tool was able to satisfactorily answer questions of a lower cognitive order, signaling a future in which the lines between human competence and artificial resources become increasingly blurred. This sheds light on a pressing issue: the need for deep reflection not only on the role of the radiologist in the face of imminent technological changes but also on professional training.

The fact that ChatGPT came close to passing a radiology exam is not to be ignored. On the contrary, it could be a harbinger of the inevitable evolution and improvement of AI algorithms. The ability of such tools to learn and improve is inherent to their programming, suggesting that it is only a matter of time before they overcome their current limitations. That perspective is supported by international studies, such as some that demonstrated the ability of ChatGPT to pass similar tests^(2,3)^, highlighting the rapid progression of these technologies.

Although the emergence of AI applications in the field of radiology will inevitably change the profession, that is not cause for reactionary alarm. It has the potential to be a valuable ally for professionals who know how to use it, not only improving diagnostic accuracy but also optimizing the production of reports and the efficiency of physician work. By automating repetitive tasks and providing unbiased second opinions, AI will free radiologists to perform the more complex tasks required in medical practice, such as clinical decisionmaking and patient care.

Given the scenario outlined above, it is imperative that medical training, teaching, and residency programs embrace new AI applications. Medical education must evolve to equip future professionals with the knowledge and skills necessary to leverage the capabilities of AI, teaching them to use these new tools effectively and ethically. The integration of AI into medical training will prepare radiologists to act not simply as technology operators but as leaders in implementing innovative solutions that improve healthcare.

The satisfactory performance of ChatGPT on radiology exams serves as a powerful reminder that we are entering a new era in medicine. We believe that AI will not replace radiologists; rather, it will provide an opportunity to redefine and enrich radiology practice. Medical entities responsible for awarding specialist degrees also need to review the topics and types of questions covered in their exams. The future demands that such questions address, for example, aspects of the role that radiologists play in multidisciplinary meetings.

As we prepare for the changes to come, refuting AI as a partner in the advancement of medicine and diagnostic imaging approaches naiveté. Although the journey ahead will be complex and full of challenges, including ethical ones, it cannot be averted.
